# Impact of healthy aging on active bacterial assemblages throughout the gastrointestinal tract

**DOI:** 10.1080/19490976.2021.1966261

**Published:** 2021-08-30

**Authors:** Kerstin Schütte, Christian Schulz, Ramiro Vilchez-Vargas, Riccardo Vasapolli, Frederike Palm, Bianca Simon, Dirk Schomburg, Anke Lux, Robert Geffers, Dietmar H. Pieper, Alexander Link, Peter Malfertheiner

**Affiliations:** a Department of Gastroenterology, Hepatology and Infectious Diseases, Otto-von-Guericke University Magdeburg, Magdeburg, Germany; b Department of Internal Medicine and Gastroenterology, Niels-Stensen-Kliniken Marienhospital Osnabrück, Bischofsstr. 1, Osnabrück, Germany; c Department of Internal Medicine 2, University Hospital, Munich, Germany; d Institute of Biometry and Medical Informatics, Otto-von-Guericke University Magdeburg, Magdeburg, Germany; e GMAK Research Group, Helmholtz Centre for Infection Research, Braunschweig, Germany; f Microbial Interactions and Processes (MINP) Research Group, Helmholtz Centre for Infection Research, Braunschweig, Germany

**Keywords:** Healthy aging, gut microbiota, nutrition, physical fitness, microbiome

## Abstract

The adaption of gut microbiota (GM) throughout human life is a key factor in maintaining health. Interventions to restore a healthy GM composition may have the potential to improve health and disease outcomes in the elderly. We performed a comprehensive characterization of changes in the luminal and mucosa-associated microbiota composition in elderly compared with younger healthy individuals. Samples from saliva and feces, and biopsies from the upper and lower gastrointestinal tract (UGIT, LGIT), were collected from 59 asymptomatic individuals grouped by age: 40–55, 56–70, and 71–85 years). All underwent anthropometric, geriatric, and nutritional assessment. RNA was extracted and reverse-transcribed into complementary DNA; the V1–V2 regions of 16S ribosomal RNA genes were amplified and sequenced. Abundances of the taxa in all taxonomic ranks in each sample type were used to construct sample-similarity matrices by the Bray–Curtis algorithm. Significant differences between defined groups were assessed by analysis of similarity. The bacterial community showed strong interindividual variations and a clear distinction between samples from UGIT, LGIT, and feces. While in saliva some taxa were affected by aging, this number was considerably greater in UGIT and was subsequently higher in LGIT. Unexpectedly, aging scarcely influenced the bacterial community of feces over the age range of 40–85 years. The development of interventions to preserve and restore human health with increased age by establishing a healthy gut microbiome should not rely solely on data from fecal analysis, as the intestinal mucosa is affected by more significant changes, which differ from those observed in fecal analyses.

## Introduction

Gut microbiota (GM) adapt to human life in a continuous, dynamic process that is essential for maintaining health.

In early childhood (up to 3 years of age) the composition of GM goes through a phase of high vulnerability; it stabilizes in adulthood and returns to vulnerability with advancing age (over 60 years). During the individual’s entire life span, GM is exposed to numerous influencing factors that include diet, lifestyle, medications, intercurrent diseases, and the process of aging itself.^1^^, 2^

Aging is a complex and multifactorial process that results in a broad variety of phenotypes ranging from frailty in some older people, while others experience healthy aging with no or little disability.

In the elderly, shifts in GM composition may additionally contribute to gastrointestinal and systemic morbidities. Interactions with GM are reported for all organ systems, including the gut-brain and gut-muscle axes, and this may contribute to frailty.^3–6^ Frailty and the environmental setting make an impact on dietary patterns, and this is reflected in GM composition.^7^

Aging is accompanied by significant changes in lifestyle, such as decreased locomotion, nutritional changes, chronic consumption of medications, and, in some cases, change in residential status. It is unclear whether these factors lead to microbiome shifts that further influence aging-related deterioration, or whether the specific individual microbiome itself regulates some of the physiological responses to environmental change with aging.^8^

Several studies have addressed this question by investigating the changes of the GM composition in elderly populations.^7,9–11^ Most such studies lacked a careful assessment of critical influencing factors such as dietary habits, medications, fitness or frailty. Furthermore, studies related to GM and aging have so far focused on oral or fecal microbiota compositions, but did not include the mucosa-adherent GM at specific sites of the gastrointestinal tract. Recent studies have shown significant changes between mucosal and luminal GM composition in the upper and lower gastrointestinal tract,^12,13^ and these appear likely to have an important effect on the interpretation of local and systemic GM-related functions.

We therefore performed a comprehensive analysis of changes in lumina- and mucosa-associated GM composition in healthy elderly individuals compared with younger persons.

## Results

In this prospective study, 59 healthy individuals were analyzed. There were no relevant age-dependent differences in physical performance, nutrition, or body composition.

Their clinical characteristics and performance in multidimensional prognostic index (MPI) stratified by age group are summarized in Table 1. No significant differences in MPI were detected, confirming that the individuals included were healthy. Participants in age group C exhibited an increased SPMSQ score in the dementia screen; however, this was still within the range characteristic of normal mental functioning.

**Table 1.** Characterization of the cohort with respect to age, multidimensional prognostic index (MPI) and its subunits, BIA measurement and anthropometric measurements and nutritional intake. Group A, 40–55 years old; group B, 56–70 years old; group C, 71–85 years old. Median and Interquartile Range (IQR) (in brackets) are shown. *p* values were calculated with the Kruskal-Wallis test. “ns” denotes no significant difference. Abbreviations are defined in Materials and Methods

	Group A (*N* = 19)	Group B (*N* = 24)	Group C (*N* = 16)	*p*
Age (median(IQR)	50 (8)	64 (7)	75 (5)	–
male/female	11/8	9/15	8/8	ns
**MPI and subunits of MPI**				
MPI (median(IQR))	0.06 (0.13)	0.06 (0.11)	0.06 (0.17)	ns
ADL (median(IQR))	6.00 (0.00)	6.00 (0.00)	6.00 (0.00)	ns
IADL (median(IQR))	8.00 (0.00)	8.00 (0.00)	8.00 (0.00)	ns
SPMSQ (median(IQR))	1.00 (1.00)	0.00 (0.00)	1.00 (1.75)	0.02
ESS (median(IQR))	20.00 (0.00)	20.00 (0.00)	20.00 (0.00)	ns
CIRS_IS (median(IQR))	1.15 (0.15)	1.19 (0.25)	1.23 (0.31)	ns
CIRS_CI (median(IQR))	0.00 (0.00)	0.00 (0.75)	0.00 (1.00)	ns
MNA (median(IQR))	24.00 (4.00)	25.50 (2.50)	25.50 (2.38)	ns
**Anthropometric and BIA measurements**				
BMI (median(IQR))	23.60 (4.50)	25.43 (4.85)	26.60 (5.30)	ns
ECF (%)				
(median(IQR))	42.25 (5.82)	47.50 (5.95)	48.30 (6.20)	< 0.001
TBW (median(IQR))	38.05 (16.50)	33.00 (12.30)	32.10 (9.10)	ns
BCM (median(IQR))	29.55 (13.55)	23.40 (8.90)	21.20 (5.10)	0.04
PA (median(IQR))	6.55 (1.28)	5.60 (1.25)	5.50 (1.10)	0.001
**Key nutritional data**				
**Macronutrient compounds**				
Carbohydrates. [g/day] (median(IQR))	229 (151)	215 (100)	247 (80)	ns
Protein [g/day] (median(IQR))	80 (48)	69 (39)	96 (41)	ns
Fat [g/day]				
(median(IQR))	116 (76)	99 (32)	127 (57)	ns
**Micronutrient compounds**				
Hexadeca-tetraenoic acid [mg/day]				
(median(IQR))	0.004 (0.02)	0 (0)	0 (0)	0.001
Docosadienoic acid [mg/day]				
(median(IQR))	0.01 (0.06)	0 (0)	0 (0)	0.001
Docosatrienoic acid [mg/day]				
(median(IQR))	0.2 (0.8)	0.06 (0.05)	0.07 (0.04)	0.006

As expected, BCM decreased significantly (*p* = .001) with age, as did PA (*p* = .001) and cell rate (*p* = .001).^14^ In contrast, an increase of extracellular fluid (*p* < .001) was observed with increasing age, which explains the observation that BMI did not show statistically relevant differences with age (Table 1).

Concerning dietary intake, the median amounts of the macronutrients fat, protein, and carbohydrates did not differ significantly between age groups. There were no age-dependent differences in intake of most micronutrients; however, there were decreases in intake of hexadecatetraenoic acid, docosadienoic acid, and docosatrienoic acid with increasing age (Table 1 and Supplementary Table 1).

### Overall bacterial community structure across the human gastrointestinal tract

Transcriptionally active bacterial communities from saliva (*n* = 42), upper gastrointestinal tract (UGIT; *n* = 96), lower gastrointestinal tract (LGIT; *n* = 121) and feces (*n* = 55) from 59 individuals were characterized after exclusion of samples in which the minimum sequencing depth was not reached (Supplementary Table 2). After sequencing and rarefying to the minimum sequencing depth, 10,034 sequences from each sample were retrieved. Taxonomic annotation revealed the presence of 14,424 unique phylotypes belonging to 22 different phyla and 430 genera (Supplementary Table 3).

The general bacterial community showed strong interindividual variations and a clear distinction between samples from UGIT, LGIT, and feces (Supplementary Figure 1). There were no statistically significant differences in the pairwise Bray–Curtis similarities of the overall bacterial community between or within the age groups for any of the taxonomic ranks analyzed. However, the average similarity between samples clearly decreased along the gastrointestinal tract, from 52.9 ± 15.1 (saliva), to 46.2 ± 16.8 (UGIT), to 35.4 ± 20.1 (LGIT) and was 41.5 ± 11.8 in feces, referring to all samples in each region independently of aging.

PERMANOVA and ANOSIM showed that during aging the bacterial communities evolved differentially along the different regions of the gastrointestinal tract (Supplementary Table 4). Bacterial communities in saliva differed between groups A (40–55 years) and B (56–70 years), independently of the taxonomic rank considered (phylum through to phylotype); however, those of the oldest individuals (Group C, 71–85 years) did not differ significantly from those of the younger groups.

In contrast, in the UGIT, significant differences in the overall bacterial communities from phylum to genus level were observed only between groups B and C in PERMANOVA. Differences between groups A and B were just observed at phylotype level (with a *p* value of 0.01). In the LGIT, no statistically significant differences between any of the groups were observed for higher taxonomic ranks (phylum, class, and order) in PERMANOVA. However, at family and genus level, significant differences between groups A and B, and also between groups A and C, were evident. In feces, PERMANOVA showed only differences between groups B and C, similar to the findings in UGIT, for phylum to family taxonomy ranks, while ANOSIM revealed no differences at any taxonomic rank.

*Diversity estimators and phylotype frequencies throughout the human gastrointestinal tract and changes with aging*

**Figure 1.**
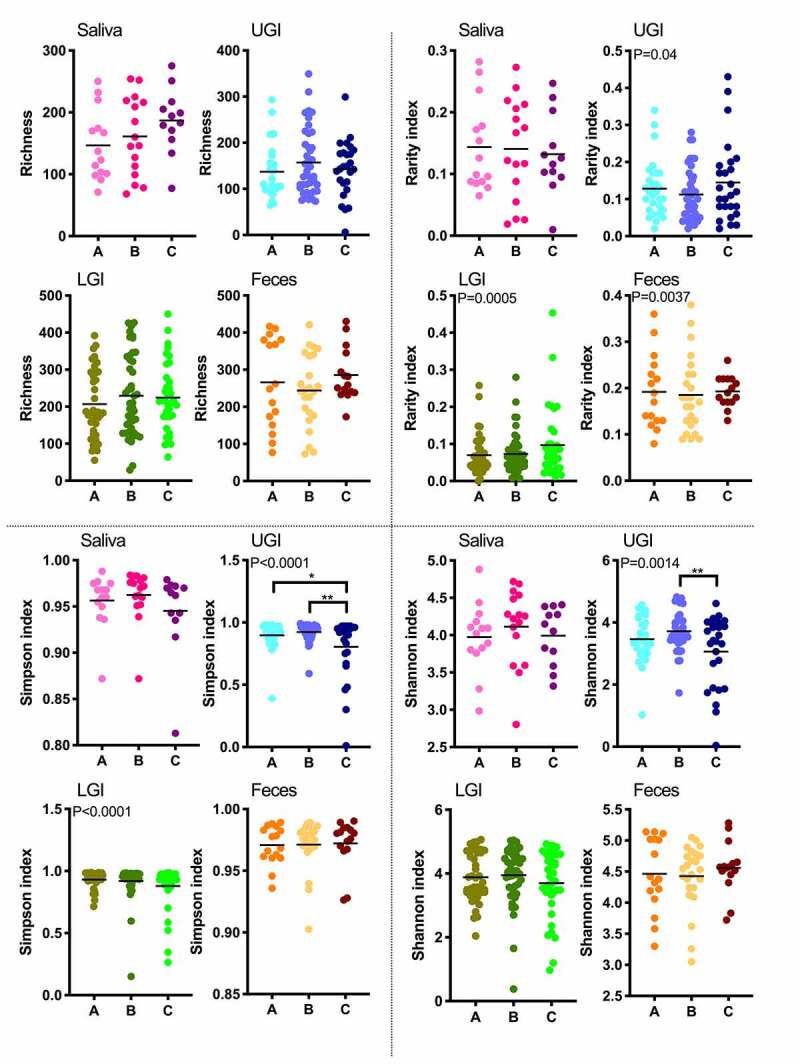
Diversity indices in each region and in each age group. Richness (upper left), rarity index (top right), Simpson index (lower left) and Shannon index (lower right)

Aging did not show any effect on the richness (defined as the total number of different phylotypes per sample) or relative rarity index in saliva, UGIT, LGIT, or feces. However, there was a significant decrease in diversity in UGIT communities as indicated by the Simpson and Shannon indices: lower values were observed in Group C than in the other groups (Figure 1). When the variances between groups were compared, no significant age-related differences regarding the variance in richness were observed (Bartlett’s test, *p* < .05). However, the variance of the relative rarity index increased with age in UGIT and LGIT communities, while it decreased in fecal communities. An increase in the variances with age was also detected for the Simpson index in UGIT and LGIT, and the Shannon index in UGIT communities (Figure 1).

* *p* < .05, ** *p* < .01. Bars indicate the mean value.If the variance was significantly different within the region and between the age groups, the *p* value from Bartlett’s test is indicated at top left.

**Figure 2.**
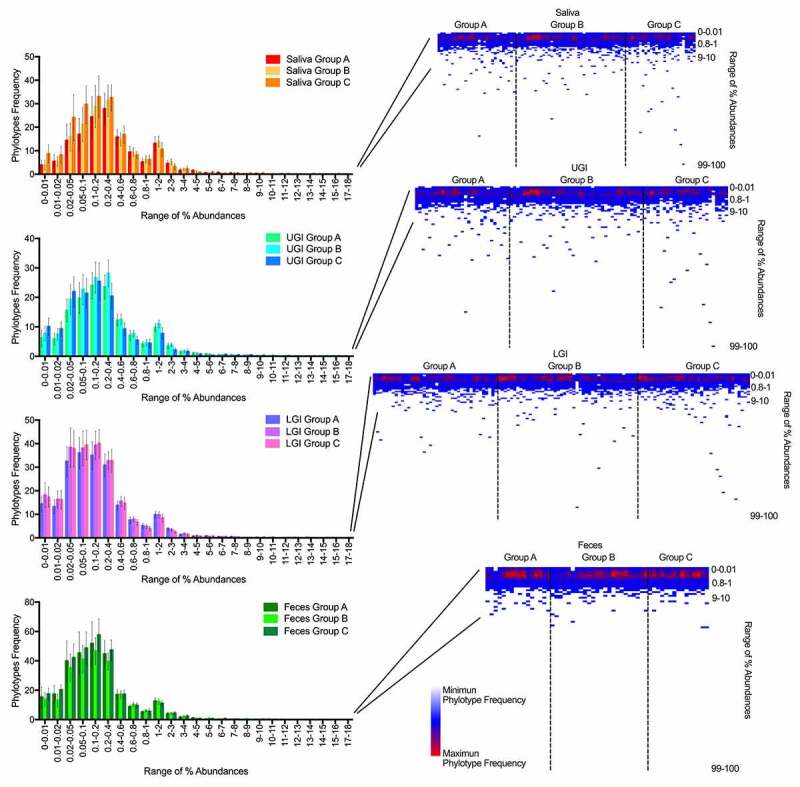
Left: Number of phylotypes in certain predefined abundance ranges (0% to 18%) for each site of the GI tract (top to bottom) and age. The abundance ranges are shown on the X axis and the total number of phylotypes detected in each range on the Y axis. Right: Heatmap showing the number of phylotypes covering the entire range from 0% to 100% of relative abundances in different regions (top to down) and in the age groups (left to right)

The number of phylotypes that were present in the community in the relative abundance range 0–18% is shown in Figure 2. Overall, the frequency distribution at which phylotypes of this abundance range were observed followed the same pattern in saliva, UGIT, LGIT, and feces. The majority of phylotypes were present in the community in a relative abundance of 0.02–0.6% in either saliva, upper GI, lower GI, or feces (Figure 2) and covered (mean ± standard deviation) (67 ± 10)% of the richness in saliva (67 ± 8)% in UGIT, (71 ± 6)% in LGIT and (73 ± 6)% in feces. Phylotypes present with a relative abundance below 10% covered roughly 99% of the richness. However, phylotypes with an abundance higher than 10% were observed with a higher frequency in older individuals, especially in saliva, UGIT and LGIT, but not in fecal samples (Figure 2). This is in agreement with the increased variance in diversity indices in older individuals, described above.

### Age-related changes in microbial communities

While in saliva a few taxa were affected by aging, this number was considerably greater in the UGIT and greater still in the LGIT, where more differences were found between aging groups. Unexpectedly, aging was only associated with minor variations in the bacterial community in feces during the entire age range of 40–85 years (Supplementary Table 5).

**Figure 3.**
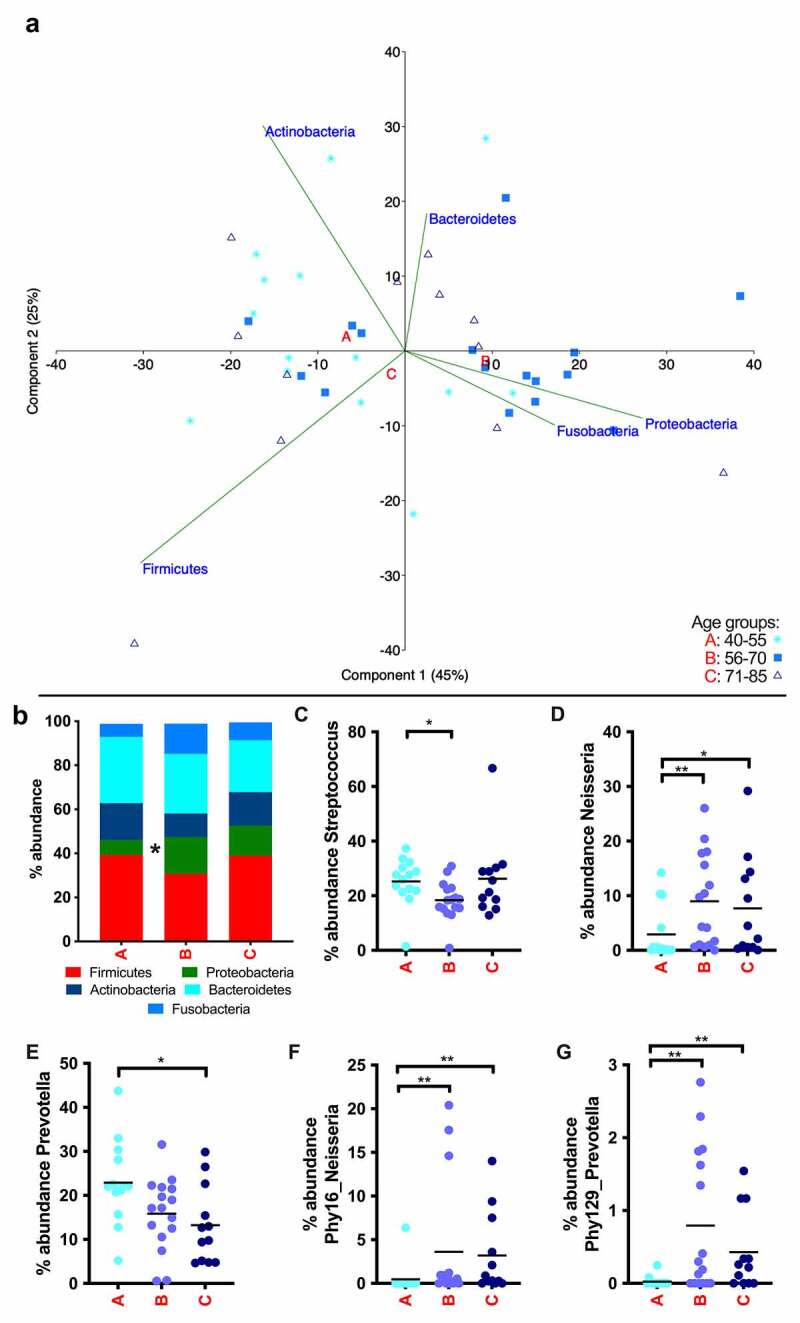
Summary of the taxa affected by age in saliva. (a) Results of principal component analysis shown by age group and phylum. Group A (40–55 years) in light blue stars, group B (56–70 years) in middle blue squares and group C (71–85 years) in dark blue triangles. Letters A, B and C are located in the centroids of the correspondent aging group. (b) Overview of the bacterial community composition by age group and phylum. (c)–(g) Age-dependent changes in abundance of the most representative taxa by age group. Bars indicate the mean value.

The three most abundant phyla in the saliva were Firmicutes, with *Streptococcus* as the most prominent genus, Bacteroidetes, with *Prevotella* as the most prominent genus and Proteobacteria, with *Neisseria* as the most prominent genus, making up roughly 80% of the total bacterial community (Figure 3). The significant differences in the overall community structure between the age groups, as evidenced by PERMANOVA and ANOSIM, can be partially explained by the abundances in those genera. The genus *Streptococcus* showed similar relative abundances in groups A and C, but was observed in lower abundance in group B (Figure 3g). Also, the genus *Neisseria* showed a strong increase in relative abundance between groups A and B and only a slight decrease between groups B and C (Figure 3e). The genus *Prevotella* was the only one detected in saliva that diminished progressively in its relative abundance with age, although the difference only became significant when groups A and C were compared (Figure 3c). At phylotype level an increase of certain sequence types during aging was detected. For instance, Phy129 (*Prevotella pallens*) increased from 0.02% in group A to 0.8% in group B and 0.4% in group C, and Phy16 (*Neisseria subflava/perflava*) increased from 0.5% in group A to 3.6% in group B and 3.2% in group C (Supplementary Table 5).

* *p* < .05, ** *p* < .01.

**Figure 4.**
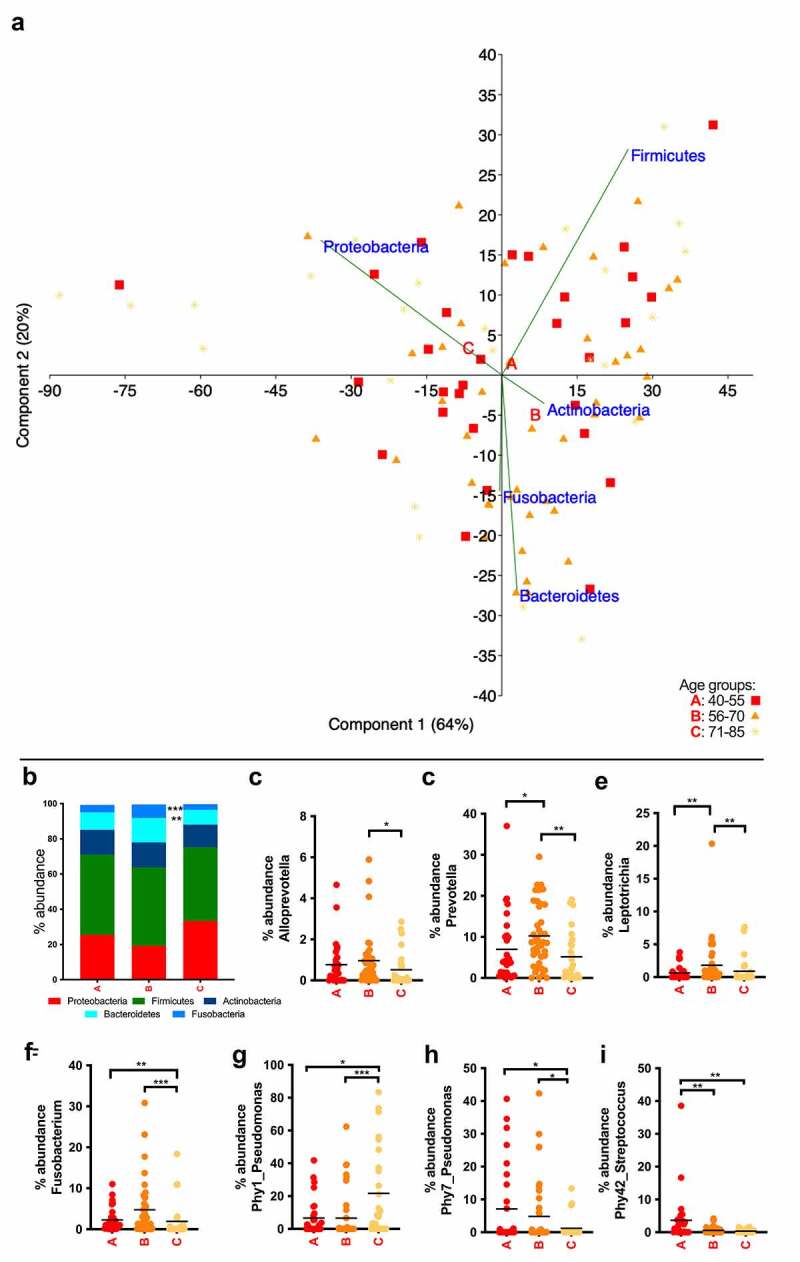
Summary of the taxa affected by age in mucosa from the upper GI tract. (a) Principal-component analysis at phylum level. Group A (40–55 years) in red squares, group B (56–70 years) in orange triangles and group C (56–70 years) in yellow stars. Letters A, B and C are located in the centroids of the correspondent aging group. (b) Overview at phylum level of the bacterial community composition. (c)–(i), age-dependent changes in abundance of the most representative taxa. Bars indicate the mean value.

In the UGIT more taxa were found to be associated with aging than in saliva (Figure 4). At phylum level, Bacteroidetes and Fusobacteria had the highest relative abundance in the middle age group B compared to groups A and C (Figure 4b). Accordingly, genera *Alloprevotella* and *Prevotella*, belonging to Bacteroidetes, and *Leptotrichia* and *Fusobacterium*, belonging to Fusobacteria, showed the highest relative abundance in group B compared to both other groups (Figure 4c, 4d, 4e and 4f). The genus *Neisseria* also had the highest relative abundance in group B (Supplementary Table 5). At phylotype level, Phy42 (*Streptococcus mitis/pneumoniae*), among others (Supplementary Table 5), was negatively influenced by aging decreasing from A to B, being less or even negligibly abundant in oldest individuals. Intriguingly, in the UGIT, two phylotypes belonging to *Pseudomonas* were found to be strongly age-related but in opposite senses: while Phy1 (*Pseudomonas sp*.) increased with age (Figure 4h), Phy7 (*Pseudomonas sp*.) diminished (Figure 4i).

* *p* < .05, ** *p* < .01, *** *p* < .0001.

**Figure 5.**
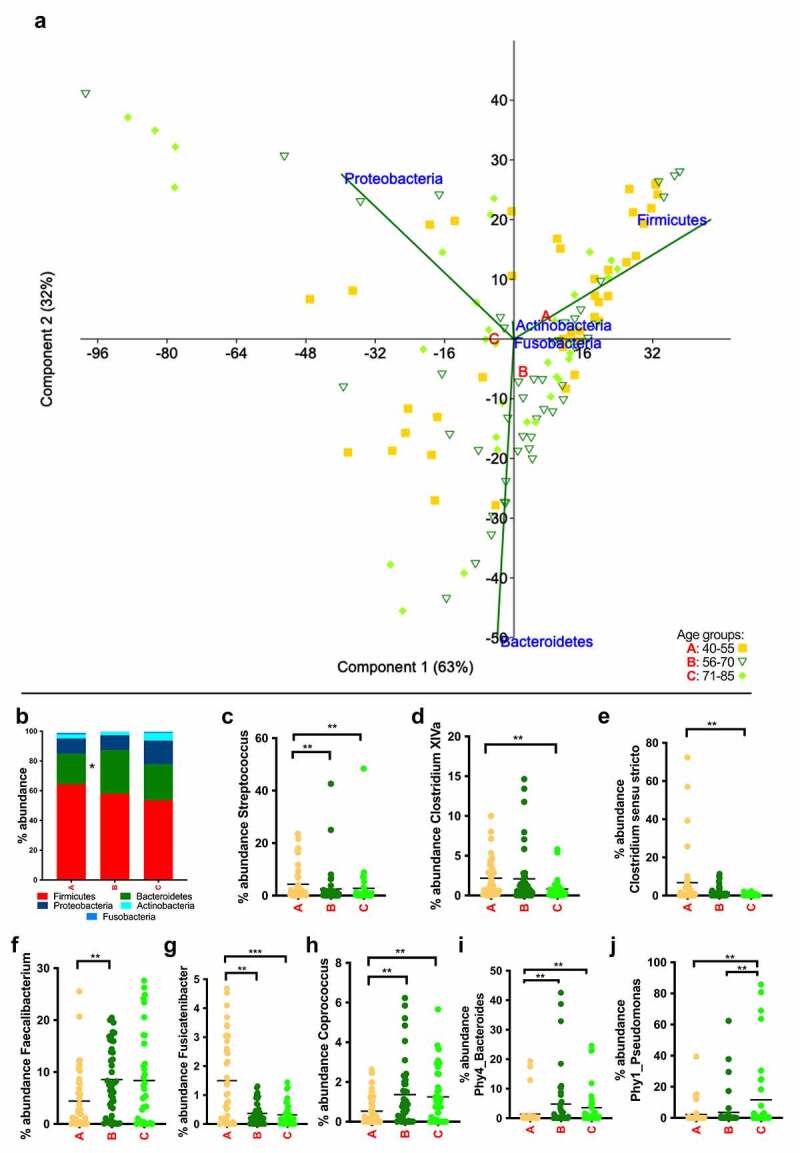
Summary of the taxa affected by age in mucosa from the lower GI tract. (a) Principal-component analysis at phylum level. Group A (40–55 years) in yellow squares, group B (56–70 years) in dark green triangles and group C (71–85 years) in light green diamonds. Letters A, B and C are located in the centroids of the correspondent aging group. (b) Overview at phylum level of the bacterial community composition (different colors correspond to different phyla). (c)–(j) Age-dependent changes in abundance of the most representative taxa. Bars indicate the mean value

As mentioned above, more effects of age on the bacterial communities were found in the LGIT (Supplementary Table 5). As previously published,^12^ bacterial communities from the UGIT differed strongly from those in the LGIT. At phylum level, only Bacteroidetes increased in relative abundance in group B compared with group A (Figure 5b). Interestingly, Firmicutes did not change significantly in abundance between age groups. However, when the bacterial communities were analyzed at genus level, all genera that were significantly affected by age belonged to Firmicutes, suggesting that Firmicutes genera changed differently during aging. In fact, *Streptococcus* or *Fusicatenibacter* diminished in relative abundance in a similar manner (from 4% and 1.5%, respectively, in group A to roughly 2.5% and 0.3%, respectively, in both of groups B and C), while *Faecalibacterium* and *Coprococcus* increased in relative abundance (from 4.4% and 0.5%, respectively, in group A to roughly 8% and 1.3%, respectively, in groups B and C). *Clostridium* XIVa diminished from roughly 2% in groups A and B to 0.8% in group C, and *Clostridium sensu stricto* decreased progressively in relative abundance with age (Figure 5c to 5h). At phylotype level two phylotypes stood out of 23 phylotypes detected as being affected (Figures 5i and 5j, and Supplementary Table 5). Phy1 (*Pseudomonas sp*.) and Phy4 (*Bacteroides dorei*), among others, increased strongly with age.

* *p* < .05, ** *p* < .01, *** *p* < .0001.

**Figure 6.**
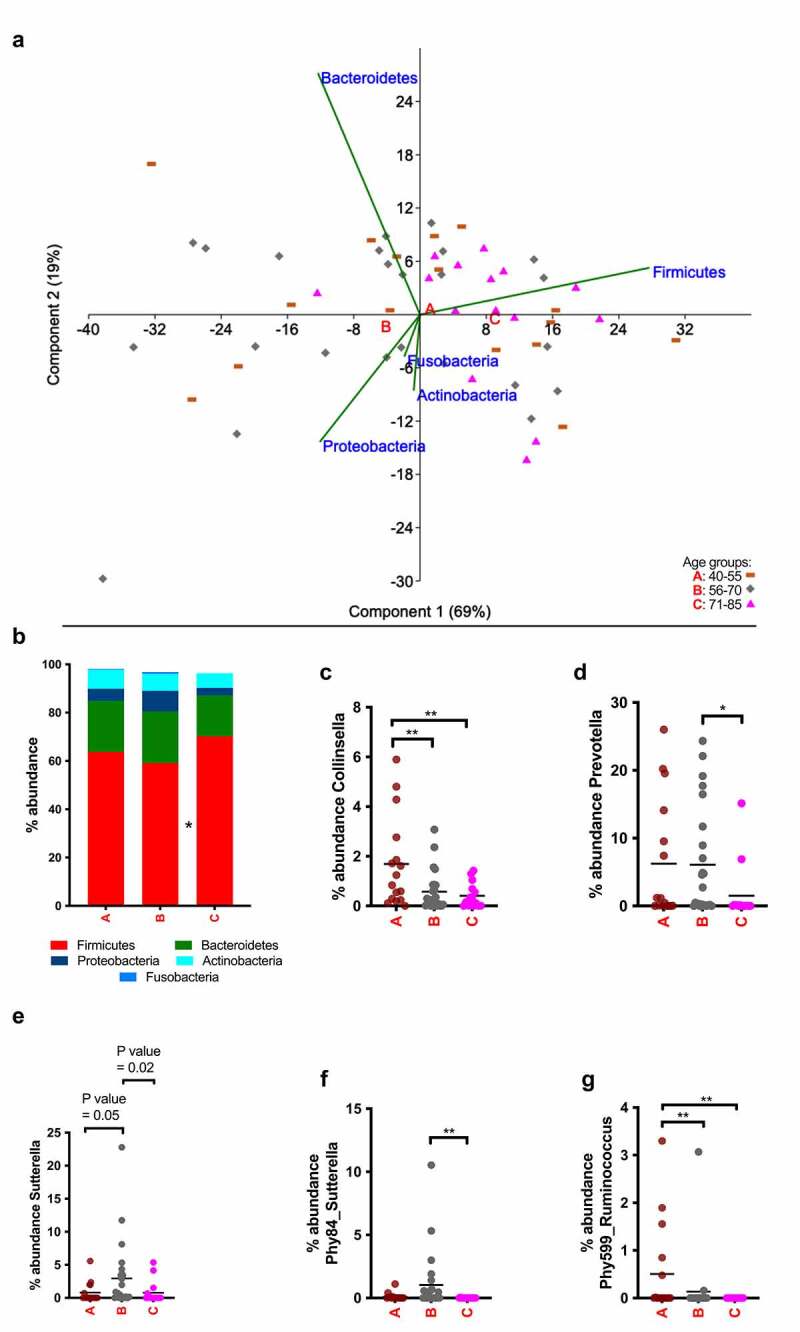
Summary of the taxa affected by age in fecal samples. (a) Principal-component analysis at phylum level. Group A (40–55 years) in brown bars, group B (56–70 years) in gray diamonds and group C (71–85 years) in pink triangles. Letters A, B and C are located in the centroids of the correspondent aging group. (b) Overview at phylum level of the bacterial community composition (different colors correspond to different phyla). (c)–(f) Age-dependent changes in abundance of the most representative taxa. Bars indicate the mean value

The fecal bacterial community was only slightly influenced by age. At phylum level only Firmicutes increased significantly in group C compared with group B (Figure 6b); however, for lower taxonomic ranks no specific taxa belonging to Firmicutes increased significantly in relative abundance. *Collinsella*, belonging to Actinobacteria, diminished drastically in relative abundance in the older individuals (>55 years old) compared to group A, and *Prevotella*, belonging to Bacteroidetes, decreased in relative abundance in individuals above 70 years old (Figures 6c and 6d).

* *p* < .05. ** *p* < .01.

### Differences in abundances of the most widely considered probiotic taxa at different sites

Overall, out of the 10,034 phylotypes detected in the whole cohort, only 105 with a median abundance of 0% (a minimum of 0% and a maximum of 13%) belonged to *Lactobacillus* spp.; 64 phylotypes with a median abundance of 0.07% (a minimum of 0% and a maximum of 14%) belonged to *Bifidobacterium* spp. and only 5 with a median abundance of 0% (range 0–0.2%) to *Akkermansia* spp. In contrast, 854 phylotypes with a median abundance of a 5.2% (range 0–85%) belonged to *Streptococcus* spp., 720 with a median abundance of 2.3% (range 0–46%) to *Prevotella* spp. and 1064 with a median abundance of 0.7% (range 0–47%) to the family Ruminococcaceae. This suggests that the species considered as eubiotics are not dominant in the microbial community of the gastrointestinal tract. In addition, with the exception of *Bifidobacterium* and only in feces, none had a median abundance higher than 0.12% in saliva, upper GIT, lower GIT or feces in any of the age groups. *Bifidobacterium* in feces was detected with a median abundance of 5.2%, 4.7% and 2.6 in groups A, B, and C, respectively, but no significant differences regarding aging were depicted. It is remarkable that the majority of subjects in all age groups showed undetectable levels of *Lactobacillus* spp. and *Akkermansia* spp. in saliva, UGIT and LGIT, *Lactobacillus* spp. was detected in feces with a neglectable median abundance (0.06% in group A, 0.04% in group B and 0.01% in group C).

## Discussion

We found age-dependent changes in the structure of active bacterial assemblages in saliva and mucosal samples of healthy individuals along the gastrointestinal tract. These changes were observed at all taxonomic levels from phyla to phylotypes, but were present with a distinct pattern at different sites along the GIT. The greatest changes were found in mucosal samples from the lower GIT.

In the transition from younger age (group A) to age above 70 years (group C), we found a decrease in relative abundance of *Prevotella* (belonging to the phylum Firmicutes) and an increase in relative abundance of *Neisseria* (belonging to the phylum Proteobacteria) in saliva.

Similar changes have not previously been reported, but their functional significance remains uncertain. An analysis of age-related variation of bacterial communities at different body sites suggested that age had a marginal impact on the structure of microbiota in the oral cavity.^15^ Others have reported that healthy elderly above 70 years harbor an oral microbiota of a diversity higher than in individuals with morbidities.^16^ Elderly persons living in a nursing home were found to harbor a lower relative abundance of Bacteroidetes (and Fusobacteria), and higher relative abundance of Actinobacteria and Firmicutes.^16^ In our cohort of healthy individuals, however, aging did not affect the abundance of these phyla, but we observed an increase of Proteobacteria from age group A (40–55 years old) to group B (56–70 years old).

In the mucosal microbiota, the most conspicuous shifts occurred in the group aged >70 years. Differences between age groups were observed in a higher number of taxa compared with saliva. Mucosa from LGIT was more affected in relation to aging compared to the mucosa from UGIT, and in addition, the taxa affected differed between UGIT and LGIT mucosal samples.

Streptococci are physiologically more abundant in the upper gastrointestinal tract than in the lower GI tract, where their abundance is almost negligible compared with other taxa.^12^ In line with those findings, we observed the same in the whole aging cohort. However, while the relative abundances of genus *Streptococcus* were not influenced by aging in the upper GI tract, we observed that the relative abundances significantly diminished after age 55 years in the lower GI tract comparing group B and C to group A. The precise role of *Streptococci* in the lower GI tract is still uncertain. However, lower abundances of genus *Streptococcus* might increase the risk of potential infection with Candida albicans, since Streptococci inhibit hypha formation in C. albicans, forcing this yeast to remain in a planktonic state.^17^

A decrease of butyrate-producers such as *Clostridium* XIVa with aging in biopsies from LGIT would indicate an important weakening of fundamental intestinal functions associated with deterioration of mucosal barrier integrity in individuals of older age.

*Prevotella* decreases in abundance in later phases of life in saliva, in the mucosa of the UGIT and also in feces. *Prevotella* spp. possess a high genetic diversity and contribute to polysaccharide breakdown. Their loss in abundance leads to a decrease in saccharolytic bacteria and an increase in proteolytic bacteria. Similar findings with aging were reported earlier.^18^

A decrease in abundance of genus *Fusicatenibacter* in in the mucosa of the LGIT may further contribute to the intestinal loss of saccharogenic activity which is a characteristic feature of the microbiome in the aging process.^19^ Age-related microbial changes, which consist of an increase in proteolytic bacteria and a decrease in saccharolytic bacteria, are associated with sarcopenia and longevity.^18^

The gut microbiota signatures have up to now been most extensively studied in feces. We observed minor changes with advancing age, while others report that fecal microbiota composition becomes less diverse and more dynamic in advanced age,^9^ with a distinct core microbiota composition significantly differing from that of younger people^20^ and strong influences from the environment and nutrition.^7^ Reports on an increase in Proteobacteria with aging are conflicting.^11,20–25^ In our study – except for Firmicutes, with a significant increase in the oldest age group – differences among age groups at phylum level were not observed in feces.

It has been hypothesized that Clostridia play a key role in modulating gut homeostasis during the whole lifespan.^26^ We found a higher abundance in feces of the order Clostridiales in the oldest group. In this respect data in the literature are conflicting: some confirm an increase, while others report a decrease in the number of anaerobes in feces above the age of 65 years.^20,26–28^ *Clostridium sensu stricto* and *Clostridium XIVa* strongly diminish with aging in mucosal samples from LGIT, while no such shifts were observed in feces in our study.

In supercentenarians, who are supposed to be an ideal model of “healthy” by maintaining the core profile of young individuals, Bacteroidaceae, Lachnospiraceae, and Ruminococcaceae decrease in their cumulative relative abundances. This is “balanced” by the increase in subdominant “eubiotic” species including the family Christensenellaceae, and the genera *Akkermansia* and *Bifidobacterium*.^10^ These changes at the metabolic level would suggest a positive impact on immunomodulation, protection against inflammation, and a healthy metabolic homeostasis.^10^

We did not observe such changes in our cohort, either in feces or in mucosa from the lower GIT. However, we observed an increase of Ruminococcaceae, where group A showed lower abundances than groups B and C. In addition, genera claimed to have a eubiotic effect such as *Bifidobacterium, Lactobacillus* or *Akkermansia* were found in low abundances and were not influenced in their abundances by aging.

Studies on the age-dependent changes of alpha-diversity in fecal microbiota composition remain inconclusive for various reasons. These include differences in the methodologies used, but most essentially in the selection of different elderly populations with and without comorbidities of various types.^2,29,30^ Frailty was certainly most strongly associated with alpha diversity in these studies.^2,30,31^ The comparisons of diversity estimators in our study suggest an increase in the variance of various diversity measures during aging, mainly in the mucosal UGI and LGI communities. In contrast to previously published studies, we studied metabolically active bacteria by evaluating bacterial RNA instead of DNA. This might explain why differences in fecal microbiota profiles were more pronounced in earlier studies than in ours.

Our study, with the limitation of the relatively small number of healthy subjects who agreed to full participation in the comprehensive clinical work-up, has the value of including in the analysis saliva, fecal samples, and in addition mucosal samples from UGIT and LGIT. In this way, we were able to demonstrate that aging effects differ between mucosal samples and luminal samples. The mucosal bacterial communities with their interaction with the gut barrier might be more closely linked to the host side of aging, possibly also being influenced by the host genetics and immune system.

Obviously, in healthy aging saliva and mucosa associated bacterial microbiota are slightly altered in their composition, whereas fecal bacterial communities are less strongly affected. Differences might occur later in life if a reduction in physical fitness or changes in other extrinsic factors (i.e. dieatary habits, consumption of medications) occur.

As we analyzed healthy individuals with physiological age-dependent changes in body composition but without relevant age-dependent differences in fitness and nutrition and in the absence of comorbidities, these factors did not bias our findings. Differences in bacterial communities with aging published from analyses in feces might to a large extent be a consequence of extrinsic factors like nutrition and environment.

Differences in participants analyzed, different nutrition patterns between the published cohorts and different regions from where the participants were recruited further impede comparisons of age-related changes between published studies and our data.^2^

We aimed at a description of a GM consortium in the elderly that might eventually allow to identify gut microbiota modulating interventions beneficial in the prevention of frailty in elderly. With this, we did not succeed. However, we clearly show that the development of such interventions should not solely rely on data from fecal analysis, as the intestinal mucosa is affected by more significant changes. In particular, we show that taxa commonly in use as probiotics, including *Lactobacillus gasseri, Bifidobacterium adolescentis* and *Akkermansia muciniphila*, do not significantly differ in abundance between age groups.^32,33^ Our findings may lead to further investigations on how to intervene actively in supporting healthy and “autonomous” aging by interventional gut microbiota modulation.

Numerous products, including nutraceuticals, pre- and probiotics, are already commercially promoted with the claim to preserve and restore human health by establishing a healthy gut microbiome with aging; however, at present sufficient evidence for this is lacking.

For fully exploiting and understanding GM adaptation in the elderly one needs to consider that aging is a process of substantial intra- and interindividual variations. In the aging process, an accumulation of deficits takes place over the entire lifespan, with dynamic interactions between an individual´s genome, epigenome, and external factors (exposome).^34–36^ Because of the nature of our study, we cannot report the dynamics of a healthy GM consortium during aging. Only with the help of longitudinal follow-up studies it will be possible to provide the answers on the role of the aging on microbiota composition (gut integrity and role of low-grade inflammation) and on microbiota function and are desirable for the future. It is not appropriate to extrapolate the overall physiological effects of specific taxa or their metabolites when the analysis is restricted to singular time points and biological compartments.^37^

## Summary and conclusion

In our study, the role of aging on the GM composition along the entire human gastrointestinal tract has been extended by the analysis of the metabolic active bacterial communities adhering at the mucosa while previous studies were limited to analyzing the effect of aging on the microbiota composition in feces and saliva.

However, the complex host–microbiota interactions need to be understood better before therapeutic interventions to maintain a healthy state can be developed.^38^

## Materials and methods

### Cohort

Asymptomatic individuals were recruited within the EMGASTA study (DRKS-ID: DRKS00009737), a large prospective study focused on research into GM profiles in health and disease. The study was approved by the local Ethics Committee and government authorities and was conformed to current Good Clinical Practice guidelines and the Declaration of Helsinki. Written informed consent was obtained from each participant. Subjects were considered asymptomatic if they were free from any functional gastrointestinal disease, tumor disease, metabolic or cardiovascular disease requiring therapy, or neurodegenerative disease. Individuals did not report any antibiotic intake within the previous 8 weeks or regular therapy with proton pump-inhibitors (PPI). Individuals were grouped into sub-cohorts by age (Group A, 40–55 years; Group B, 56–70 years; Group C, 71–85 years).

### Clinical and nutritional assessment

All individuals underwent a clinical examination, and medical history, including history of medication, was recorded. For further assessment, a comprehensive geriatric assessment was performed and the multidimensional prognostic index (MPI) was calculated. This index includes information on cohabitation status, medication use, activities of daily living (ADL), the instrumental activities of daily living scale (IADL), the short portable mental status questionnaire (SPMSQ), the Exton-Smith scale (ESS), the cumulative illness rating scale (CIRS) with its subscales rating comorbidities (CIRS_CI) and their severity (CIRS_IS) and the mini nutritional assessment (MNA). These add up to a well-validated score to predict mortality in older subjects.^39–46^

Bioelectrical impedance analysis (BIA) provides an option to obtain information on the nutritional status of patients, as malnutrition is associated with changes in body composition. BIA is a noninvasive, reproducible method and has been validated for the assessment of body composition and nutritional status in various patient populations.^47,48^ The phase angle (PA) is a function of both resistance and reactance; it reflects the proportion of cellular mass, the integrity of cell membranes and hydration status, and it represents a biological marker of cellular health.^49^ PA declines with age and sarcopenia.^50,51^ Study participants underwent anthropometric measurements (height, weight, body mass index (BMI), thigh circumference and mid-upper-arm circumference (MUAC)) and a BIA measurement including phase-angle and computed analysis of derived parameters including extracellular water (ECW), intracellular water (ICW), total body water (TBW), fat mass (FM) and body-cell mass (BCM) in addition to clinical and laboratory assessment. Measurement was performed with a BIACORPUS RX 4000 BIA analyzer (MEDI CAL Healthcare GmbH, Karlsruhe, Germany). BIA was conducted with the patients lying supine on a bed with their legs apart, arms not touching the torso. All evaluations were conducted using the four-surface standard electrode (tetra polar) technique on the hand and foot. R and Xc were measured directly in Ω at 50 kHz. One assessment of R and Xc was made. PA was calculated by the equation PA = (R/Xc) x (180/π). Other analyses were performed with the software BodyComp V8.5 (MEDI CAL Healthcare GmbH, Karlsruhe, Germany).

For the evaluation of nutritional pattern, individuals were asked to answer the German Epic Food Frequency Questionnaire 2 (FFQ2), which is a self-administered, semiquantitative, and simple questionnaire comprising 102 food items, originally developed and validated to measure consumption of specific foods over the previous years.^52^ For each food item, participants were asked about frequency of consumption of a predefined portion size. Frequency of intake was measured using a scale of 8 categories from “never,” “≤1 time per month or less” to “≥3 times per day.”

Macro- and micronutrient intakes were obtained by using the German Food Code and Nutrient Database (version II.3) and provided by the Department of Epidemiology of the German Institute of Human Nutrition, Potsdam-Rehbrucke.^53^

All statistical analyses were performed using IBM SPSS Statistics 24.0.0 (IBM Corporation, New York, NY, USA). Results for numerical data are given as mean with standard deviation; for categorical data, as absolute numbers with percentage. For comparison of categorical data, the X^2^ test was applied if the expected incidence exceeded 5; otherwise Fisher’s exact test was used. Kruskal–Wallis tests were used for checking the homogeneity of independent samples in continuous data. All statistical comparisons were two-sided, with a critical probability of α = 0.05 and without α adjustment.

### Bacterial community assessment

Samples from saliva and feces and biopsies from the stomach (corpus and antrum), duodenum, terminal ileum and colon (descending and ascending) were obtained from each participant.

All methods and procedures are described elsewhere.^12,54^ Briefly, RNA was extracted using the RNeasy kit (Qiagen) following manufacturer’s instructions with a mechanical lysis step. After DNA digestion, first-strand cDNA was synthesized with the SuperScript IV First-Strand Synthesis System (Invitrogen, Carlsbad, California, USA) and random primers, following manufacturer’s instructions. Amplicon libraries were generated by amplification of the V1-V2 region of the 16S rRNA after 20-cycle PCR using the primers 27 F and 338 R and sequenced on a MiSeq (2 × 250 bp, Illumina, Hayward, California, USA).^55,56^

FastQ files were analyzed with the dada2 package version 1.10.1, in R.^57^ Overall, 11,963,371 paired-end reads were obtained, with a minimum of 8427 and average of 37,739 per sample. Samples that did not reach 10,000 reads were discarded for downstream analysis. All samples were resampled to equal sequencing depth of 10,034 reads using the phyloseq package^58^ returning 14,424 phylotypes (Supplementary Table 2). Phylotypes were annotated to a taxonomic affiliation based on the naïve Bayesian classification^59^ with a pseudo-bootstrap threshold of 80%. The taxonomy annotation was done following the traditional taxonomy, considering the 16S rRNA gene (ribosomal dataproject release 11.5 with RDP taxonomy training set No 16). Microbial communities were analyzed systematically at different phylogenetic ranks in a sequential manner: from phylum to class, order, family, genus and phylotype. Relative abundances (%) were used for downstream analyses. Phylotypes with different abundances between groups defined *a priori* were analyzed manually by comparison with the NCBI database to define the discriminatory power of each sequence read. A species name was assigned to a phylotype when 16S rRNA gene fragments of previously described isolates of that species were ≥98% identical with the respective representative sequence read. The EcoIndR package^60^ of R was used for calculating richness, rarity index (based on the equation of Leroy^61,62^), and Simpson (1–λ) and Shannon (H’) indices. Statistical tests were performed with Prism7 (Graph Pad Software, Inc.). First, each variable of interest was subjected to a normality test using the D’Agostino & Pearson omnibus. Since most of the diversity variables returned estimates indicating normal distributions, ANOVA with multiple comparisons to analyze for differences between groups of samples and Bartlett’s test were performed to seek differences in variance between groups of samples. All *p* values were corrected by applying the Benjamini-Krieger-Yekutieli false-discovery-rate correction (desired FDR = 5%), and it was considered significant if the corrected *p* value was <0.05.

Further statistical analyses were performed with Prism 7 (Graph Pad Software, Inc.) or Past 3.^63^ A dendrogram was built by using the sample-similarity matrix based on the Bray–Curtis algorithm using MegaX. Differences between age groups (see “Cohort”) in saliva, upper (biopsies from antrum, corpus, and duodenum) and lower (biopsies from terminal ileum, ascending colon and descending colon) GI and feces were evaluated with PERMANOVA and ANOSIM (9999 permutations). Groups were considered statistically different if *p* < .05. Bacterial communities from each group were characterized from phylum to phylotype (see above); differences in distribution of taxa among the three groups were calculated by the Kruskal–Wallis test for multiple comparisons and *p* values were corrected by the two-stage linear step-up Benjamini-Krieger-Yekutieli procedure (desired FDR = 5%).^64^ The abundance of taxa between groups was considered significantly different if the corrected *p* value was below 0.05. For the Kruskal–Wallis test only those taxa with a mean of 1% of abundance for levels from phylum to family and a mean of 0.5% of abundance for the ranks genus and phylotypes were considered.

The phylotype frequency per sample was calculated by dividing the percentage of abundances into 108 intervals (0–0.01%, 0.01–0.02%, 0.02–0.05%, 0.05–0.1%, 0.1–0.2%, 0.2–0.4%, 0.4–0.6%, 0.6–0.8%, 0.8–1% and thereafter in steps of 1% up to 100%). For each interval, the total number of phylotypes was calculated and expressed as a percentage of overall abundance.

## Supplementary Material

Supplemental Material

## Abbreviations used in this paper:

ADL, activities of daily living; ANOSIM, analysis of similarities; BCM, body-cell mass; BIA, bioelectrical impedance analysis; BMI, body mass index; CIRS, cumulative illness rating scale; ECW, extracellular water; ESS, Exton-Smith scale; FFQ, food frequency questionnarie; FM, fat mass; GM, gut microbiota; GI, gastrointestinal; ICW, intracellular water; IADL, instrumental activities of daily living; LGIT, lower gastrointestinal tract; MNA, mini nutritional assessment; MPI, multidimensional prognostic index; MUAC, mid-upper-arm circumference; PA, phase angle; PERMANOVA, permutational multivariate analysis of variance; rRNA, ribosomal RNA; SPMSQ, short portable mental status questionnaire; TBW, total body water; UGIT, upper gastrointestinal tract
