# Supplementary material for: Impact of healthy aging on active bacterial assemblages throughout the gastrointestinal tract
Source: Gut Microbes. 2021 Aug 30;13(1):1966261. doi: 10.1080/19490976.2021.1966261 (PMC8409759; doi:10.1080/19490976.2021.1966261)
Supplement: Supplemental Material [file KGMI_A_1966261_SM7046.zip › Supplementary information/ST5_P_values.pdf]

Rank	Taxon	Phylum affiliation	Saliva findings	Mean A	Mean B	Mean C	q value	P Value	
Phylum	Proteobacteria	Proteobacteria	A-B	6.81	16.7	13.7	0.0067	0.0032	**
Class	Betaproteobacteria	Proteobacteria	A-B	3.33	10.3	8.49	0.0033	0.0032	**
Class	Betaproteobacteria	Proteobacteria	A-C	3.33	10.3	8.49	0.0121	0.023	*
Order	Lactobacillales	Firmicutes	A-B	28.1	19.4	27.6	0.0095	0.0045	**
Order	Neisseriales	Proteobacteria	A-B	3.23	9.35	8.26	0.0056	0.0053	**
Order	Neisseriales	Proteobacteria	A-C	3.23	9.35	8.26	0.0131	0.025	*
Family	Streptococcaceae	Firmicutes	A-B	25.2	18.4	26.3	0.0142	0.0068	**
Family	Neisseriaceae	Proteobacteria	A-B	3.23	9.35	8.26	0.0056	0.0053	**
Family	Neisseriaceae	Proteobacteria	A-C	3.23	9.35	8.26	0.0131	0.025	*
Genus	Streptococcus	Firmicutes	A-B	25.2	18.4	26.2	0.0142	0.0068	*
Genus	Neisseria	Proteobacteria	A-B	2.91	8.97	7.69	0.0068	0.0033	**
Genus	Neisseria	Proteobacteria	A-C	2.91	8.97	7.69	0.0398	0.0379	*
Genus	Prevotella	Bacteroidetes	A-C	22.9	15.8	13.2	0.0209	0.0099	*
Phylotype	Phy129_Prevotella	Bacteroidetes	A-B	0.0235	0.794	0.429	0.0024	0.0023	**
Phylotype	Phy129_Prevotella	Bacteroidetes	A-C	0.0235	0.794	0.429	0.0069	0.0131	*
Phylotype	Phy110_Unclassified_Prevotellacea	Bacteroidetes	A-C	0.0513	0.453	1.44	0.0122	0.0058	*
Phylotype	Phy17_Fusobacterium	Fusobacteria	A-B	1.11	3.6	2.43	0.0151	0.0072	*
Phylotype	Phy16_Neisseria	Proteobacteria	A-B	0.464	3.61	3.19	0.0053	0.0102	**
Phylotype	Phy16_Neisseria	Proteobacteria	A-C	0.464	3.61	3.19	0.0053	0.006	**
Phylotype	Phy124_Actinomyces	Actinobacteria	A-C	0.531	0.0455	0.493	0.0066	0.0126	**
Phylotype	Phy124_Actinomyces	Actinobacteria	B-C	0.531	0.0455	0.493	0.0002	0.0002	***

UGI

Rank	Taxon	Phylum affiliation	findings	Mean A	Mean B	Mean C	q value	P Value	
Phylum	Bacteroidetes	Bacteroidetes	B-C	9.99	13.9	8.4	0.0095	0.0045	**
Phylum	Fusobacteria	Fusobacteria	B-C	4.16	7.65	3.2	0.0002	0.0001	***
Class	Bacteroidia	Bacteroidetes	A-B	9.41	13.3	7.97	0.0415	0.0396	*
Class	Bacteroidia	Fusobacteria	B-C	9.41	13.3	7.97	0.0081	0.0038	**
Class	Fusobacteriia	Fusobacteria	B-C	4.16	7.65	3.2	0.0002	0.0001	***
Class	Betaproteobacteria	Proteobacteria	A-B	3.42	4.63	2.8	0.0152	0.029	*
Class	Betaproteobacteria	Proteobacteria	B-C	3.42	4.63	2.8	0.0098	0.0094	**
Order	Coriobacteriales	Actinobacteria	B-C	0.543	1.07	0.494	0.0186	0.0088	*
Order	Bacteroidales	Bacteroidetes	A-B	9.41	13.3	7.97	0.0415	0.0396	*
Order	Bacteroidales	Bacteroidetes	B-C	9.41	13.3	7.97	0.0081	0.0038	**
Order	Fusobacteriales	Fusobacteria	A-B	4.16	7.65	3.2	0.0422	0.0574	*
Order	Fusobacteriales	Fusobacteria	A-C	4.16	7.65	3.2	0.0422	0.0602	*
Order	Fusobacteriales	Fusobacteria	B-C	4.16	7.65	3.2	0.0002	0.0001	***
Order	Neisseriales	Proteobacteria	A-B	2.64	4.16	2.48	0.0479	0.0456	*
Order	Neisseriales	Proteobacteria	B-C	2.64	4.16	2.48	0.0225	0.0107	*
Family	Coriobacteriaceae	Actinobacteria	B-C	0.543	1.07	0.494	0.0186	0.0088	*
Family	Prevotellaceae	Bacteroidetes	B-C	8.16	11.4	6.65	0.0084	0.004	**
Family	Clostridiales_Incertae_Sedis_XI	Firmicutes	A-C	1.16	0.793	0.266	0.0137	0.026	*
Family	Clostridiales_Incertae_Sedis_XI	Firmicutes	B-C	1.16	0.793	0.266	0.0137	0.0256	*
Family	Fusobacteriaceae	Fusobacteria	A-C	2.26	4.74	1.9	0.0079	0.015	**
Family	Fusobacteriaceae	Fusobacteria	B-C	2.26	4.74	1.9	0.0003	0.0003	***
Family	Leptotrichiaceae	Fusobacteria	A-B	1.9	2.91	1.29	0.0104	0.0197	*
Family	Leptotrichiaceae	Fusobacteria	B-C	1.9	2.91	1.29	0.0006	0.0006	***
Family	Neisseriaceae	Proteobacteria	A-B	2.64	4.16	2.48	0.0479	0.0456	*
Family	Neisseriaceae	Proteobacteria	B-C	2.64	4.16	2.48	0.0225	0.0107	*
Family	Helicobacteraceae	Proteobacteria	A-B	4.12	0.571	4.48	0.0304	0.0145	*
Genus	Alloprevotella	Bacteroidetes	B-C	0.77	0.961	0.514	0.0186	0.0089	*
Genus	Prevotella	Bacteroidetes	A-B	6.98	10.2	5.16	0.0164	0.0312	*
Genus	Prevotella	Bacteroidetes	B-C	6.98	10.2	5.16	0.003	0.0028	**
Genus	Parvimonas	Firmicutes	A-C	1.12	0.779	0.242	0.0049	0.0094	**
Genus	Parvimonas	Firmicutes	B-C	1.12	0.779	0.242	0.0049	0.0084	**
Genus	Fusobacterium	Fusobacteria	A-C	2.26	4.74	1.9	0.0079	0.015	**
Genus	Fusobacterium	Fusobacteria	B-C	2.26	4.74	1.9	0.0003	0.0003	***
Genus	Leptotrichia	Fusobacteria	A-B	0.639	1.79	0.897	0.0097	0.0185	**
Genus	Leptotrichia	Fusobacteria	B-C	0.639	1.79	0.897	0.0034	0.0033	**
Genus	Neisseria	Proteobacteria	B-C	2.56	4.01	2.36	0.0208	0.0099	*
Genus	Helicobacter	Proteobacteria	A-B	4.12	0.571	4.48	0.0304	0.0145	*
Phylotype	Phy87_Prevotella	Bacteroidetes	A-B	0.395	0.642	0.0859	0.0394	0.0375	*
Phylotype	Phy87_Prevotella	Bacteroidetes	B-C	0.395	0.642	0.0859	0.0215	0.0102	*
Phylotype	Phy129_Prevotella	Bacteroidetes	A-B	0.137	0.574	0.388	0.0041	0.002	**
Phylotype	Phy25_Gemella	Firmicutes	A-C	1.58	2.07	0.779	0.0476	0.0453	*
Phylotype	Phy25_Gemella	Firmicutes	B-C	1.58	2.07	0.779	0.0045	0.0021	**
Phylotype	Phy42_Streptococcus	Firmicutes	A-B	3.63	0.552	0.363	0.0012	0.0011	**
Phylotype	Phy42_Streptococcus	Firmicutes	A-C	3.63	0.552	0.363	0.002	0.0037	**
Phylotype	Phy52_Streptococcus	Firmicutes	A-B	0	1.62	0	0.0087	0.0133	**
Phylotype	Phy52_Streptococcus	Firmicutes	B-C	0	1.62	0	0.0087	0.0165	**
Phylotype	Phy102_Streptococcus	Firmicutes	A-B	0.319	0.599	0.216	0.0204	0.0097	*
Phylotype	Phy122_Streptococcus	Firmicutes	A-C	0	0.464	0.636	0.0122	0.0117	*
Phylotype	Phy122_Streptococcus	Firmicutes	B-C	0	0.464	0.636	0.0124	0.0236	*
Phylotype	Phy140_Streptococcus	Firmicutes	A-C	0.0055	0.0017	1.24	0.0151	0.0288	*
Phylotype	Phy140_Streptococcus	Firmicutes	B-C	0.0055	0.0017	1.24	0.0036	0.0035	**
Phylotype	Phy195_Streptococcus	Firmicutes	A-B	1.31	0.0277	0.000383	0.0118	0.0224	*
Phylotype	Phy195_Streptococcus	Firmicutes	A-C	1.31	0.0277	0.000383	0.0073	0.007	**
Phylotype	Phy301_Streptococcus	Firmicutes	A-C	0	0	0.58	<0.0001	<0.0001	****
Phylotype	Phy301_Streptococcus	Firmicutes	B-C	0	0	0.58	<0.0001	<0.0001	****
Phylotype	Phy115_Parvimonas	Firmicutes	A-C	0.887	0.53	0.112	0.0046	0.0088	**
Phylotype	Phy115_Parvimonas	Firmicutes	B-C	0.887	0.53	0.112	0.0045	0.0043	**
Phylotype	Phy43_Veillonella	Firmicutes	B-C	0.748	0.687	0.391	0.0294	0.014	*
Phylotype	Phy17_Fusobacterium	Fusobacteria	A-C	1.48	2.86	0.832	0.034	0.0324	*
Phylotype	Phy17_Fusobacterium	Fusobacteria	B-C	1.48	2.86	0.832	0.0006	0.0003	***
Phylotype	Phy90_Fusobacterium	Fusobacteria	A-B	0.0663	0.651	0.521	0.0115	0.0055	*
Phylotype	Phy46_Leptotrichia	Fusobacteria	A-B	0.229	0.727	0.453	0.0134	0.0254	*
Phylotype	Phy46_Leptotrichia	Fusobacteria	B-C	0.229	0.727	0.453	0.0049	0.0046	**
Phylotype	Phy16_Neisseria	Proteobacteria	A-B	0.811	3.18	1.61	0.0089	0.0169	**
Phylotype	Phy16_Neisseria	Proteobacteria	B-C	0.811	3.18	1.61	0.0089	0.0129	**
Phylotype	Phy1_Pseudomonas	Proteobacteria	A-C	6.66	6.56	21.6	0.0114	0.0217	*
Phylotype	Phy1_Pseudomonas	Proteobacteria	B-C	6.66	6.56	21.6	0.0003	0.0003	***
Phylotype	Phy7_Pseudomonas	Proteobacteria	A-C	7.1	4.82	1.17	0.0115	0.022	*
Phylotype	Phy7_Pseudomonas	Proteobacteria	B-C	7.1	4.82	1.17	0.0115	0.0169	*

LGI

Rank	Taxon	Phylum affiliation	findings	Mean A	Mean B	Mean C	q value	P Value	
Phylum	Bacteroidetes	Bacteroidetes	A-B	20.7	29.4	24.3	0.0127	0.006	*
Class	Bacteroidia	Bacteroidetes	A-B	20.6	29.4	24.3	0.0123	0.0059	*
Class	Bacilli	Firmicutes	A-B	4.86	3	4.22	0.0045	0.0022	**
Order	Bacteroidales	Bacteroidetes	A-B	20.6	29.4	24.3	0.0123	0.0059	*
Order	Lactobacillales	Firmicutes	A-B	4.63	2.65	2.92	0.0015	0.0014	**
Order	Lactobacillales	Firmicutes	A-C	4.63	2.65	2.92	0.0068	0.0129	**
Order	Erysipelotrichales	Firmicutes	A-C	4.08	5.62	2.14	0.0159	0.0303	*
Order	Erysipelotrichales	Firmicutes	B-C	4.08	5.62	2.14	0.0008	0.0008	***
Family	Streptococcaceae	Firmicutes	A-B	4.47	2.51	2.76	0.0015	0.0014	**
Family	Streptococcaceae	Firmicutes	A-C	4.47	2.51	2.76	0.0068	0.013	**
Family	Clostridiaceae_1	Firmicutes	A-C	6.74	1.62	0.274	0.002	0.0009	**
Family	Peptostreptococcaceae	Firmicutes	A-C	2.13	3.02	0.314	0.0079	0.015	**
Family	Peptostreptococcaceae	Firmicutes	B-C	2.13	3.02	0.314	0.0024	0.0023	**
Family	Ruminococcaceae	Firmicutes	A-B	7.48	11.6	12.2	0.0103	0.0165	*
Family	Ruminococcaceae	Firmicutes	A-C	7.48	11.6	12.2	0.0103	0.0197	*
Family	Erysipelotrichaceae	Firmicutes	A-C	4.08	5.62	2.14	0.0159	0.0303	*
Family	Erysipelotrichaceae	Firmicutes	B-C	4.08	5.62	2.14	0.0008	0.0008	***
Genus	Streptococcus	Firmicutes	A-B	4.32	2.51	2.76	0.0018	0.0017	**
Genus	Streptococcus	Firmicutes	A-C	4.32	2.51	2.76	0.0079	0.015	**
Genus	Clostridium_sensu_stricto	Firmicutes	A-C	6.74	1.62	0.273	0.0018	0.0009	**
Genus	Anaerostipes	Firmicutes	A-B	2.05	0.867	3.58	0.0082	0.013	**
Genus	Anaerostipes	Firmicutes	B-C	2.05	0.867	3.58	0.0082	0.0156	**
Genus	Clostridium_XlVa	Firmicutes	A-C	2.18	2.1	0.814	0.0087	0.0041	**
Genus	Coprococcus	Firmicutes	A-B	0.527	1.36	1.25	0.0075	0.0071	**
Genus	Coprococcus	Firmicutes	A-C	0.527	1.36	1.25	0.0099	0.0188	**
Genus	Fusicatenibacter	Firmicutes	A-B	1.5	0.369	0.321	0.0011	0.002	**
Genus	Fusicatenibacter	Firmicutes	A-C	1.5	0.369	0.321	0.0002	0.0002	***
Genus	Roseburia	Firmicutes	A-B	1.25	1.96	1.36	0.0145	0.0069	*
Genus	Intestinibacter	Firmicutes	B-C	1.88	2.67	0.281	0.007	0.0033	**
Genus	Faecalibacterium	Firmicutes	A-B	4.41	8.57	8.37	0.0084	0.004	**
Genus	Holdemanella	Firmicutes	A-B	0.4	2.4	0.455	0.0486	0.0463	*
Genus	Holdemanella	Firmicutes	B-C	0.4	2.4	0.455	0.0314	0.015	*
Phylotype	Phy66_Corynebacterium	Actinobacteria	A-B	0.0167	0.0314	1.54	0.0378	0.0541	*
Phylotype	Phy66_Corynebacterium	Actinobacteria	A-C	0.0167	0.0314	1.54	0.0003	0.0002	***
Phylotype	Phy66_Corynebacterium	Actinobacteria	B-C	0.0167	0.0314	1.54	0.0378	0.0435	*
Phylotype	Phy71_Collinsella	Actinobacteria	A-B	0.00673	0.289	0.664	0.0021	0.001	**
Phylotype	Phy4_Bacteroides	Bacteroidetes	A-B	1.38	4.81	3.55	0.005	0.0095	**
Phylotype	Phy4_Bacteroides	Bacteroidetes	A-C	1.38	4.81	3.55	0.005	0.0066	**
Phylotype	Phy5_Bacteroides	Bacteroidetes	A-C	0.605	1.61	2.4	0.0288	0.0137	*
Phylotype	Phy11_Bacteroides	Bacteroidetes	B-C	0.657	1.68	0.996	0.013	0.0062	*
Phylotype	Phy85_Clostridium	Firmicutes	A-B	2.71	0	0	0.0034	0.004	**
Phylotype	Phy85_Clostridium	Firmicutes	A-C	2.71	0	0	0.0034	0.0065	**
Phylotype	Phy119_Clostridium	Firmicutes	B-C	0.508	0.265	0.00554	0.0133	0.0063	*
Phylotype	Phy86_Anaerostipes	Firmicutes	A-B	0.435	0.0237	0.804	<0.0001	<0.0001	****
Phylotype	Phy86_Anaerostipes	Firmicutes	B-C	0.435	0.0237	0.804	0.0005	0.0009	***
Phylotype	Phy47_Clostridium_XlVa	Firmicutes	A-B	1.03	0.00731	0.00166	0.0009	0.0008	***
Phylotype	Phy47_Clostridium_XlVa	Firmicutes	A-C	1.03	0.00731	0.00166	0.0013	0.0024	**
Phylotype	Phy10_Ruminococcus	Firmicutes	A-C	0.703	1.02	4.45	0.0133	0.0064	*
Phylotype	Phy48_Ruminococcus2	Firmicutes	A-B	0.323	1.44	0.039	0.0269	0.0212	*
Phylotype	Phy48_Ruminococcus2	Firmicutes	A-C	0.323	1.44	0.039	0.0269	0.0256	*
Phylotype	Phy48_Ruminococcus2	Firmicutes	B-C	0.323	1.44	0.039	<0.0001	<0.0001	****
Phylotype	Phy24_Lachnospiraceae	Firmicutes	A-C	0.083	0.00177	0.96	0.0373	0.0355	*
Phylotype	Phy24_Lachnospiraceae	Firmicutes	B-C	0.083	0.00177	0.96	0.016	0.0076	*
Phylotype	Phy14_Lachnospiraceae	Firmicutes	B-C	0.157	0.00509	1.35	0.0043	0.002	**
Phylotype	Phy72_Lachnospiraceae	Firmicutes	A-B	0.548	0.00687	0.295	0.0113	0.0054	*
Phylotype	Phy8_Lachnospiraceae	Firmicutes	A-B	2.92	0.515	0.341	0.0077	0.0037	**
Phylotype	Phy225_Faecalibacterium	Firmicutes	A-C	0.000498	0	0.709	0.0014	0.0026	**
Phylotype	Phy225_Faecalibacterium	Firmicutes	B-C	0.000498	0	0.709	0.0005	0.0004	***
Phylotype	Phy32_Clostridiales	Firmicutes	A-B	2.09	0.0184	0.0172	0.0145	0.0069	*
Phylotype	Phy13_Erysipelotrichaceae	Firmicutes	A-B	1.85	0.616	0.351	0.0021	0.002	**
Phylotype	Phy13_Erysipelotrichaceae	Firmicutes	A-C	1.85	0.616	0.351	0.0024	0.0046	**
Phylotype	Phy44_E.coli/Shigella	Proteobacteria	A-C	0.448	0.135	0.537	0.0029	0.0056	**
Phylotype	Phy44_E.coli/Shigella	Proteobacteria	B-C	0.448	0.135	0.537	0.0023	0.0022	**
Phylotype	Phy91_Enterobacteriaceae	Proteobacteria	A-C	0.599	0.127	0.303	0.0037	0.0018	**
Phylotype	Phy1_Pseudomonas	Proteobacteria	A-C	2.14	3.53	11.6	0.0018	0.0017	**
Phylotype	Phy1_Pseudomonas	Proteobacteria	B-C	2.14	3.53	11.6	0.0042	0.008	**
Phylotype	Phy7_Pseudomonas	Proteobacteria	A-B	0.594	0.567	0.237	0.0153	0.0292	*
Phylotype	Phy7_Pseudomonas	Proteobacteria	A-C	0.594	0.567	0.237	0.0019	0.0018	**
Phylotype	Phy159_Proteobacteria	Proteobacteria	A-B	0.803	0	0	0.0081	0.0104	**
Phylotype	Phy159_Proteobacteria	Proteobacteria	A-C	0.803	0	0	0.0081	0.0153	**
Phylotype	Phy216_Bacteria	Unclassified	A-B	0.897	0	0	0.0081	0.0104	**
Phylotype	Phy216_Bacteria	Unclassified	A-C	0.897	0	0	0.0081	0.0153	**

Feces

Rank	Taxon	Phylum affiliation	findings	Mean A	Mean B	Mean C	q value	P Value	
Phylum	Firmicutes	Firmicutes	B-C	63.8	59.2	70.2	0.0211	0.01	*
Class	Clostridia	Firmicutes	B-C	49.3	44.8	59.5	0.0032	0.0015	**
Order	Clostridiales	Firmicutes	B-C	49.2	44.8	59.5	0.003	0.0014	**
Family	Prevotellaceae	Bacteroides	B-C	6.7	7.04	1.73	0.009	0.0043	**
Genus	Collinsella	Actinobacteria	A-B	1.69	0.575	0.409	0.0096	0.0183	*
Genus	Collinsella	Actinobacteria	A-C	1.69	0.575	0.409	0.0096	0.0142	*
Genus	Prevotella	Bacteroidetes	B-C	6.24	6.09	1.51	0.0316	0.015	*
Phylotype	Phy599_Ruminococcus	Firmicutes	A-B	0.506	0.135	0	0.006	0.0114	*
Phylotype	Phy599_Ruminococcus	Firmicutes	A-C	0.506	0.135	0	0.0037	0.0035	**
Phylotype	Phy84_Sutterella	Proteobacteria	B-C	0.11	1.04	0.00399	0.0093	0.0045	**
