# Supplementary material for: Impact of healthy aging on active bacterial assemblages throughout the gastrointestinal tract
Source: Gut Microbes. 2021 Aug 30;13(1):1966261. doi: 10.1080/19490976.2021.1966261 (PMC8409759; doi:10.1080/19490976.2021.1966261)
Supplement: Supplemental Material [file KGMI_A_1966261_SM7046.zip › Supplementary information/Supplemental material.docx]

Supplemental material:

**Figure S1:** Group-average agglomerative hierarchical clustering of 314 samples, based on global bacterial profiles (phylotype level) along the upper and lower GI tract from 59 healthy individuals. Anatomic sites are denoted by colors: Saliva (S) in light blue; stomach antrum (A), stomach corpus (C) and duodenum (D) in dark blue; terminal ileum (Tl), ascending colon (CA) and descending colon (CD) in pink; feces (F) in red.

Supplementary Table 1: Comparison of intake of micro- and macronutrients between age groups

Supplementary Table 2: number and sites of samples for analysis of microbiota composition

Supplementary Table 3: output of phylotypes

Supplementary Table 4: Results of the paired wise comparisons between regions of the entire GI tract, considering the overall community at all taxonomy ranks by PERMANOVA and ANOSIM. Groups A: 40-55 years old, group B: 56-70 years old, group C 71-85 years old.

Supplementary Table 5: means per group of all taxa detected with correspondent p-and q-values
