# Supplementary figures and images for: Impact of healthy aging on active bacterial assemblages throughout the gastrointestinal tract
Source: Gut Microbes. 2021 Aug 30;13(1):1966261. doi: 10.1080/19490976.2021.1966261 (PMC8409759; doi:10.1080/19490976.2021.1966261)

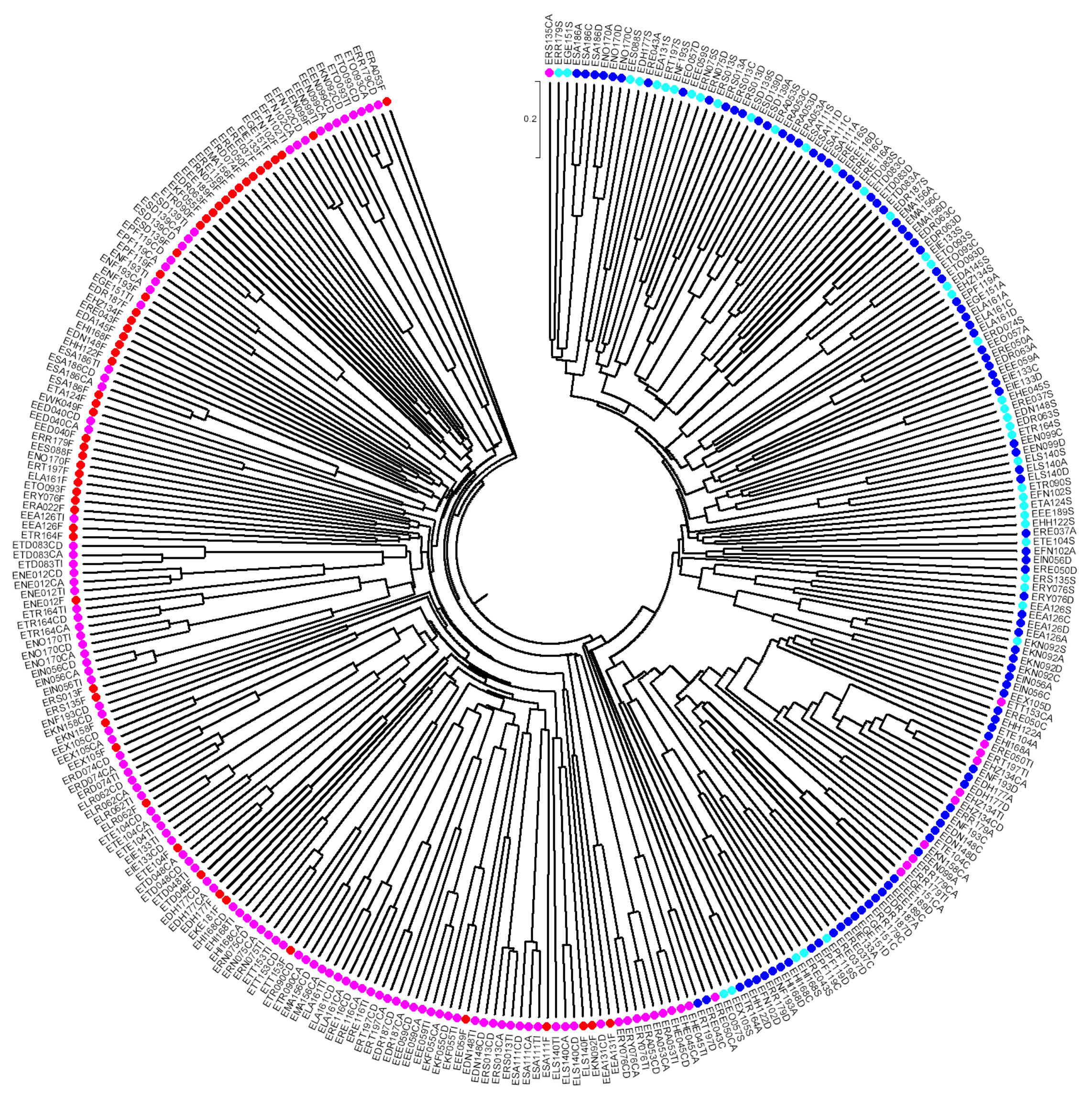

Supplement: Supplemental Material [file KGMI_A_1966261_SM7046.zip › Supplementary information/SF1_Figure_1.tiff]
